# Genetic diversity and population structure of Omani date palm assessed by SSR markers

**DOI:** 10.3389/fpls.2026.1776708

**Published:** 2026-03-02

**Authors:** Al-Ghaliya Humaid Al-Mamari, Abdullah Hamed Al-Jabri, Sean Mayes

**Affiliations:** 1 Tissue Culture and Biotechnology Research Center, Ministry of Agriculture, Fisheries and Water Resources, Barka, Oman; 2 Plant and Crop Sciences, Biosciences, University of Nottingham, Nottingham, United Kingdom

**Keywords:** AMOVA, cultivar differentiation, genetic diversity, heterozygosity, molecular markers, Nei distance, Oman, *Phoenix dactylifera*

## Abstract

Date palm (*Phoenix dactylifera* L.) is a dioecious, clonally propagated crop in which accurate genetic characterization is essential for germplasm management and breeding. This study used 12 SSR loci to quantify genetic diversity and resolve population structure within a sex-stratified Omani collection (Oman female and Oman male accessions), interpreted against a limited international reference panel (IRD) used strictly for contextual comparison. Across groups, SSR loci were highly polymorphic and captured moderate to high diversity, with mean allelic richness ranging from Na = 9.75–12.58 and expected heterozygosity from He = 0.736–0.797. Observed heterozygosity was consistently lower (Ho = 0.596–0.646), yielding positive inbreeding coefficients (Fis = 0.164–0.193). Pairwise differentiation indicated very weak sex-based subdivision within Oman (Fst = 0.021; Nei’s *D* = 0.143), whereas comparisons between Oman groups and IRD showed higher but still modest differentiation (Fst ≈ 0.057–0.059; *D* ≈ 0.508–0.534). AMOVA supported this pattern, with 92% of variation within populations and 8% among populations. Multivariate and model-based structure analyses identified clear genetic clustering overall (optimal K = 4), driven primarily by separation involving the IRD panel rather than by sex within Oman. Collectively, these findings demonstrate substantial within-Oman diversity with minimal sex-based structuring, while highlighting measurable divergence between the Omani collection and the contextual IRD reference panel.

## Introduction

Date palm (*Phoenix dactylifera* L.) is a foundation crop in arid and semi-arid regions, yet accurate identification and characterization of cultivars remain persistent challenges for germplasm conservation and breeding. Historically, cultivar discrimination has relied on morphological descriptors, including vegetative architecture and fruit traits such as size, weight, color, and flesh characteristics (Zehdi et al., 2004; Al-Ruqaishi et al., 2008). However, phenotypic traits are strongly influenced by environmental conditions and orchard management, which can obscure underlying genetic relationships (Elhoumaizi et al., 2002; Zehdi et al., 2004). In addition, morphological identification is typically impractical until palms reach reproductive maturity, often after several years, delaying reliable classification. Nomenclatural inconsistencies further complicate germplasm management, the same cultivar may be known under different names across regions, while distinct genotypes may share the same name due to similar fruit appearance (Cao and Chao, 2002).

Biochemical markers (e.g., isozymes and proteins) have been used to support cultivar identification, but their resolution is constrained by tissue specificity and developmental dependence (Bader et al., 2007). Consequently, DNA-based markers have become central to diversity assessment and fingerprinting in date palm, evolving from RFLP approaches to PCR-based systems such as RAPD, AFLP and SSRs, and more recently SNP genotyping (Johnson et al., 2009).

Among marker systems, simple sequence repeats (SSRs) (Ellegren, 2004) have been widely adopted for date palm germplasm studies because they are multi-allelic, co-dominant, and reproducible across laboratories, enabling detection of heterozygosity and discrimination among closely related cultivars (Palliyarakkal et al., 2011). SSR-based investigations have been reported for germplasm collections from several countries, including Qatar, Tunisia, Oman and Sudan (Akkak et al., 2009; Ahmed and Al-Qaradaw, 2009; Elmeer and Mattat, 2015; Al-Ruqaishi et al., 2008; Elshibli and Korpelainen, 2010).

Oman maintains a rich and historically important date palm gene pool, preserved through traditional cultivation and national germplasm collections. A biological feature of particular relevance to diversity and structure in date palm is dioecy, where male and female individuals are distinct and reproduction depends on pollen flow. This makes sex-specific representation in germplasm panels important for interpreting genetic structure and for guiding conservation and breeding decisions, especially when male genotypes are under sampled relative to females in cultivated collections.

Date palm genetic diversity is shaped by the interaction of clonal propagation and sexual reproduction. While elite cultivars are commonly maintained through offshoots, cultivation systems also include seed derived individuals and extensive pollen mediated gene flow, which can introduce new alleles and contribute to admixture over time. These features, combined with dioecy, mean that sex representation and sampling design can influence the apparent structure of germplasm panels, particularly when accessions originate from multiple regions with unequal sample sizes.

In parallel, the availability of genome resources and large-scale resequencing datasets has advanced date palm genetics from marker based fingerprinting toward genome wide inference of population history and trait discovery (Al-Mssallem et al., 2013; Hazzouri et al., 2015; Zehdi-Azouzi et al., 2015). Nevertheless, SSR markers remain valuable for cost-effective cultivar identification, germplasm curation, and first-pass assessments of diversity and relatedness across heterogeneous collections, and have been widely applied to date palm cultivar discrimination in multi-origin panels (Al-Ruqaishi et al., 2008). Accordingly, in the present study the external accessions are treated as a limited reference panel to provide contextual interpretation of broad affinities, rather than as a basis for country-level ranking of diversity.

In this study, we present an SSR-based analysis designed to characterized genetic diversity and population structure within Oman, with explicit emphasis on female and male Omani populations. To provide external context without implying a balanced global comparison, we additionally include a limited international reference panel (Iran, US, Iraq, Libya, Italy, Sudan, and France) that is treated as a contextual benchmark for interpreting broad-scale clustering patterns rather than as a basis for country-level diversity ranking which has been obtained from Institute de recherché pour le development (IRD). Specifically, our aims are to: (i) quantify and compare genetic diversity metrics between Oman female and Oman male groups; (ii) test whether sex contributes detectably to genetic structure within the Omani collection using multivariate and model-based clustering; and (iii) position Omani genetic patterns relative to the IRD reference panel in a strictly descriptive manner to aid interpretation of broader affinities.

Unlike earlier SSR surveys, this study explicitly evaluates sex-stratified structure within the Omani germplasm (female cultivars *vs* male trees) and discusses how these results can inform practical germplasm management (e.g., curating male diversity among pollen donors, accession authentication, and detection of duplicates/mislabeling). The non-Omani IRD set is used strictly as a limited contextual reference to aid interpretation of broader affinities rather than to support country-level comparisons.

## Materials and methods

### Plant material

A total of 242 date palm (*Phoenix dactylifera* L.) accessions were analyzed. The core dataset comprised 194 accessions from Oman (151 female cultivars and 43 male trees) collected from the National Date Palm Germplasm Collection at Wadi Quriyat Research Station, Bahla, Oman. Each accession was generally represented by a single tree; however, for several male genotypes, two to three replicates were sampled. Replicate samples were used for genotype quality control; for population-genetic and STRUCTURE analyses, only one representative genotype per accession was retained to avoid overweighting duplicated male genotypes. To provide external context for clustering patterns, an additional 48 non-Omani accessions from Italy (Sanremo and Bordighera), USDA-ARS collections (USA), France, Iraq, Libya, Sudan and Iran were included (**Supplementary Table 1**).

### DNA extraction and quality control

Young, mature leaf tissue was collected in the field, transported to the laboratory on ice, and stored at −80 °C until processing. Genomic DNA was extracted using the DNeasy Plant Maxi Kit (Qiagen, Hilden, Germany) according to the manufacturer’s instructions. DNA quantity and integrity were evaluated on 1% (w/v) agarose gels using λ-DNA standards (Promega) as a reference. All DNA samples were then normalized to 50 ng µL^-^¹ for SSR genotyping.

### Microsatellite amplification and fragment analysis

Genetic diversity was assessed using 12 SSR loci (**Table 1**). SSRs were selected because they provide a cost-effective, highly polymorphic and co-dominant marker system suitable for accession fingerprinting and routine germplasm curation, and they allow comparability with a large body of published date palm SSR datasets. The 12 loci were chosen from widely used date palm SSR panels reported to be robust and polymorphic across diverse germplasm, with reliable scoring on capillary electrophoresis. PCR amplification was performed using an M13-tailing protocol. Reactions were prepared from a master mix containing 10× buffer (with Mg²^+^), dNTPs, Taq polymerase (Invitrogen), and an M13-labelled universal primer, with subsequent marker-specific addition of forward and reverse primers. PCR was carried out in 96-well plates using 50 ng genomic DNA per reaction under the following cycling conditions: 94 °C for 3 min, followed by 35 cycles of 94 °C for 1 min, 50–60 °C for 1 min, and 72 °C for 2 min, with a final extension at 72 °C for 10 min.

**Table 1:** A compilation of twelve SSR markers utilized for investigating the genetic diversity of date palm accessions.

S.N	Marker name	Motif repeat	Annealing T_m_ (°)	Expected size (bp)	Source
1	mPdCIR010	(GA)_22_	52 °C	118-161	Billotte et al. (2004)
2	mPdCIR015	(GA)_21_	52 °C	120-156	Billotte et al. (2004)
3	mPdCIR016	(GA)_14_	52 °C	130-138	Billotte et al. (2004)
4	mPdCIR025	(GA)_22_	52 °C	199-231	Billotte et al. (2004)
5	mPdCIR050	(GA)_15_	52 °C	154-208	Billotte et al. (2004)
6	mPdCIR057	(GA)_20_	52 °C	251-278	Billotte et al. (2004)
7	mPdCIR093	(GA)_17_	52 °C	153-184	Billotte et al. (2004)
8	PDCAT2	TCGCTG(TC)_3_(TC)_3_T(TC)_3_T (TC)_3_T(TC)_4_TTCT GTCCCG(TC)_16_T(TC)	55 °C	166-194	Akkak et al. (2009)
9	PDCAT11	(TC)_7_ (TC)_20_	55 °C	133-155	Akkak et al. (2009)
10	PDCAT12	(CT)_19_	55 °C	145-167	Akkak et al. (2009)
11	PDCAT14	(TC)_19_ (TC)_16_	55 °C	114-145	Akkak et al. (2009)
12	PDCAT20	(GA)_29_	55 °C	290-353	Akkak et al. (2009)

The marker name, motif repeat, annealing temperature, expected product size, and original source are provided.

PCR products were first checked on 2% (w/v) agarose gels and then sized by capillary electrophoresis using the Beckman Coulter CEQ™ 8000 Genetic Analysis System (Beckman-Coulter, Fullerton, CA). For each run, 1 µL of PCR product was combined with CEQ loading solution and the CEQ-400 internal size standard. Allele sizes were called using the Beckman Coulter CEQ analysis software.

### Data scoring and statistical analysis

Alleles were scored based on fragment size and exported to an Excel-format allelic matrix. Genetic diversity statistics, including allele counts and frequencies, observed and expected heterozygosity (H_o_, H_e_), and fixation indices (Fis, Fst, Fit), were calculated using GenAlEx v6.5 (Peakall and Smouse, 2012), following Wright’s F-statistics framework (Wright, 1965). Principal Coordinates Analysis (PCoA) was conducted using Nei’s genetic distance matrices.

Genetic differentiation among populations was further evaluated using AMOVA with 999 permutations. Polymorphism Information Content (PIC) was computed in PowerMarker v3.25 (Liu and Muse, 2005). Cluster analysis was performed in DARwin v6.0 using the unweighted neighbor-joining approach (Perrier and Jacquemoud-Collet, 2019).

Population structure was inferred using STRUCTURE v2.3.4 (Pritchard et al., 2000) with K = 2–10, applying a 100,000 burn-in and 100,000 MCMC iterations per run, with 10 replicate runs for each K. The most likely number of clusters was determined using the ΔK method (Evanno et al., 2005), implemented via STRUCTURESELECTOR (Li and Liu, 2017).

Because STRUCTURE can be sensitive with limited marker numbers and unbalanced sampling, results were interpreted conservatively and cross-checked against PCoA and distance-based clustering.

## Results

### Genetic diversity at microsatellite loci

Given unequal external sample sizes, all inferences beyond Oman are treated as qualitative context rather than country-level diversity comparisons. All 12 microsatellite loci were polymorphic across the 242 date palm accessions, indicating that each marker detected measurable genetic variation in the panel (**Table 2**).

**Table 2:** Summary of microsatellite allele of date palm.

Locus name	MAF	TNA	*Na*	*N_e_*	*H_e_*	*H_o_*	*PIC*	*HWE*	*G_st_*	*I*	*F_is_*	*F_it_*	*F_st_*	*N_m_*
mPdCIR010	0.2037	27	15.333	7.013	0.854	0.785	0.892	0.105^ns^	0.037	2.235	0.081	0.121	0.043	5.576
mPdCIR015	0.1811	21	11.333	5.623	0.815	0.783	0.880	0.740 ^ns^	0.083	1.915	0.040	0.125	0.088	2.579
mPdCIR016	0.2119	15	9.000	5.462	0.816	0.579	0.848	0.000^***^	0.051	1.889	0.291	0.332	0.058	4.040
mPdCIR025	0.2675	22	11.667	5.530	0.818	0.664	0.833	0.007^**^	0.051	1.958	0.188	0.234	0.058	4.093
mPdCIR050	0.465	25	13.333	4.302	0.754	0.624	0.748	0.000^***^	0.030	1.873	0.173	0.204	0.037	6.475
mPdCIR057	0.8025	9	5.333	1.874	0.382	0.347	0.334	0.000^***^	0.089	0.814	0.091	0.178	0.095	2.370
mPdCIR093	0.4074	14	9.000	4.354	0.752	0.668	0.770	0.000^***^	0.050	1.703	0.112	0.162	0.057	4.167
PDCAT2	0.1955	24	12.333	5.621	0.821	0.808	0.862	0.216 ^ns^	0.074	1.914	0.016	0.094	0.080	2.875
PDCAT11	0.1914	22	12.667	5.589	0.816	0.629	0.885	0.000^***^	0.062	2.019	0.229	0.282	0.068	3.403
PDCAT12	0.4918	20	10.000	3.654	0.711	0.314	0.682	0.000^***^	0.050	1.604	0.559	0.585	0.059	3.960
PDCAT14	0.2366	23	14.000	6.394	0.829	0.636	0.876	0.635 ^ns^	0.045	2.099	0.233	0.273	0.052	4.538
PDCAT20	0.3477	24	12.333	4.537	0.774	0.600	0.762	0.000^***^	0.041	1.863	0.224	0.262	0.048	4.935
Mean	0.334	21.000	11.361	4.996	0.762	0.620	0.781	–	0.055	1.824	0.186	0.238	0.062	4.084

Major alleles frequency (MAF), Total Number of alleles (TNA), mean number of alleles (Na), effective number of alleles (Ne), expected heterozygosity (He), observed heterozygosity (Ho), polymorphic information content (PIC), Hardy-Weinberg Equilibrium (HWE) (ns=not, significant, **P<0.01, ***P<0.001), Shannon information index (I), gene differentiation coefficient (G_st_), F-statistics (*F_is_, F_it_* and *F_st_*) and gene flow (*N_m_*) values for 12 SSR loci analyzed in 242 P*. dactylifera* accessions.

### Allelic variation and allele frequency patterns

Substantial allelic richness was observed across loci. The total number of alleles per locus (TNA) ranged from 9 (mPdCIR057) to 27 (mPdCIR010), with a mean of 21 alleles, reflecting a generally high level of allelic diversity. Major allele frequency (MAF) values were mostly low to moderate (mean 0.334), consistent with broadly distributed allele frequencies rather than dominance by a single allele at most loci. The lowest MAF was recorded for mPdCIR015 (0.181), while mPdCIR057 showed the highest MAF (0.803), indicating markedly reduced allele evenness at this locus (**Table 2**).

### Heterozygosity and effective allele number

Expected heterozygosity (He) was generally high across loci (mean 0.762), ranging from 0.382 at mPdCIR057 to 0.854 at mPdCIR010. Observed heterozygosity (Ho) varied more widely (mean 0.620), from 0.314 (PDCAT12) to 0.808 (PDCAT2), with several loci showing lower Ho than He, consistent with a heterozygote deficit at those markers. The effective number of alleles (Ne) supported this pattern: Ne ranged from 1.874 (mPdCIR057) to 7.013 (mPdCIR010), with a mean of 4.996, indicating that most loci maintained multiple alleles at appreciable frequencies, whereas mPdCIR057 was comparatively less diverse (**Table 2**).

### Marker informativeness (PIC)

Polymorphism information content (PIC) values were high overall (mean 0.781), ranging from 0.334 (mPdCIR057) to 0.892 (mPdCIR010). Most loci exhibited PIC values above ~0.7, indicating strong discriminatory capacity, while mPdCIR057 was notably less informative relative to the remaining markers (**Table 2**).

### Hardy–Weinberg equilibrium and differentiation indices

Several loci showed statistically significant deviation from Hardy–Weinberg equilibrium, (e.g., mPdCIR050, mPdCIR057, mPdCIR093, PDCAT11, PDCAT12, PDCAT20), whereas others were non-significant (e.g., mPdCIR010, mPdCIR015, PDCAT2, PDCAT14). Across loci, genetic differentiation (Gst) values were low to moderate (mean 0.055, range 0.030–0.089), and estimated gene flow (Nm) was correspondingly moderate to high (mean 4.084), with the lowest Nm at mPdCIR057 (2.370) and the highest at mPdCIR050 (6.475) (**Table 2**).

### SSR-based genetic diversity in the date palm population

Genetic diversity was assessed using 12 SSR markers across the studied date palm groups (Oman female, Oman male, and the limited IRD female reference panel). Across all groups, the SSR dataset revealed moderate-to-high genetic variation, with consistent polymorphism reflected by multi-allelic loci and relatively high heterozygosity estimates (**Table 3**).

**Table 3:** Genetic diversity parameter among date palm population estimated using 12 SSR markers.

	N	Na	Ne	Ho	He	Fis	PA
Oman Female	146.833	12.583	4.907	0.596	0.736	0.187	3.333
Oman Male	42.250	9.750	4.843	0.617	0.753	0.164	2.667
IRD Female	47.917	11.750	5.238	0.646	0.797	0.193	4.833
Mean	–	11.361	4.996	0.620	0.762	0.181	3.611

Sample Size (N), Mean number of alleles (Na), Effective number of alleles (Ne), Observed Heterozygosity (Ho), Expected Heterozygosity (He), number of Private alleles (PA) and inbreeding coefficient (F_is_).

Mean allelic richness (Na) differed among groups. Oman females showed the highest mean allele number (Na = 12.583), followed by IRD females (Na = 11.750), while Oman males had a lower mean (Na = 9.750). Effective allele numbers (Ne), which account for allele frequency distribution, were comparatively similar across groups, ranging from 4.843–5.238. This indicates that, despite differences in total allele counts, the proportion of alleles occurring at appreciable frequencies was broadly comparable across groups (**Table 3**).

Private alleles (PA) were detected in all groups, supporting the presence of group-specific variation. The IRD female panel showed the greatest mean number of private alleles (PA = 4.833), followed by Oman females (PA = 3.333) and Oman males (PA = 2.667), suggesting that each group contributes unique allelic variants to the overall diversity observed (**Table 3**).

Observed heterozygosity (Ho) was moderate across groups (0.596–0.646) and was consistently lower than expected heterozygosity (He) (0.736–0.797). Specifically, IRD females exhibited the highest heterozygosity levels (Ho = 0.646; He = 0.797), while Oman females showed slightly lower values (Ho = 0.596; He = 0.736). Oman males displayed intermediate observed heterozygosity (Ho = 0.617) but relatively high expected heterozygosity (He = 0.753) (**Table 3**).

In all groups, positive inbreeding coefficients (Fis) were observed (0.164–0.193), consistent with a heterozygote deficit relative to Hardy–Weinberg expectations at the group level. The magnitude of Fis was broadly similar across groups, indicating that the departure from expected heterozygosity was not confined to a single population subset (**Table 3**).

### Pairwise Nei’s genetic distance and Fst among date palm populations (Oman female *vs* Oman male; contextual IRD comparison)

Pairwise comparisons based on 12 SSR loci revealed low to moderate genetic differentiation among the three date palm groups, with consistent patterns across both metrics (**Table 4**).

**Table 4:** Pairwise population matrix of Nei genetic distance (below diagonal) and Pairwise Population Fst Values (above diagonal) among date palm populations estimated from 12 microsatellite loci.

Oman female	Oman male	IRD female	
0.000	0.021	0.059	Oman female
0.143	0.000	0.057	Oman male
0.508	0.534	0.000	IRD female

below diagonal = Nei’s genetic distance (D); above diagonal = Fst.

Using Fst (above the diagonal), the lowest differentiation was observed between Oman Female and Oman Male (Fst = 0.021), indicating very limited genetic subdivision between sexes within the Omani collection. In contrast, comparisons involving the IRD Female reference panel showed higher but still modest differentiation. Oman Female *vs* IRD Female (Fst = 0.059) and Oman Male *vs* IRD Female (Fst = 0.057). Overall, these values suggest that the IRD panel is genetically distinguishable from the Omani groups, while the two Omani sex-based groups remain closely related.

The pattern was reinforced by Nei’s genetic distance (below the diagonal). The smallest distance occurred between Oman Female and Oman Male (Nei’s *D* = 0.143), consistent with strong genetic similarity between these groups. Distances between each Omani group and IRD Female were markedly larger (Nei’s *D* = 0.508 for Oman Female–IRD Female; 0.534 for Oman Male–IRD Female), indicating that the IRD panel is more divergent and forms a distinct grouping relative to the Omani accessions.

Taken together, both indices support a simple clustering pattern in which Oman Female and Oman Male cluster closely, whereas IRD Female is separated from the Omani groups, consistent with stronger genetic affinity within Oman and greater divergence between Oman and the external reference panel.

### Analysis of molecular variance of SSR variation among date palm groups

AMOVA based on 12 SSR loci showed that most genetic variation was distributed within populations, with a smaller but measurable component attributable to differences among populations (**Table 5**). Variation within populations accounted for 92% of the total molecular variance (estimated variance = 4.577), indicating that individuals within each group retain substantial allelic diversity. In contrast, variation among populations explained 8% of the total variance (estimated variance = 0.377), demonstrating a modest level of population differentiation across the defined groups.

**Table 5:** Analysis of molecular variance (AMOVA) for 12 SSR loci across date palm populations.

Source	Degree of freedom	Sum of square	MS	Estimated variance	% of variation
Among Pops	2	107.823	53.911	0.377	8%
Within Pops	483	2210.708	4.577	4.577	92%
Total	485	2318.531	–	4.954	100%

Overall, these results indicate that the sampled date palm groups are not strongly subdivided genetically, and that most diversity is maintained within populations rather than being partitioned between them. This pattern is consistent with a scenario where groups share much of their genetic background, while still showing detectable structure at the population level.

### Principal coordinates analysis

**Figure 1:**
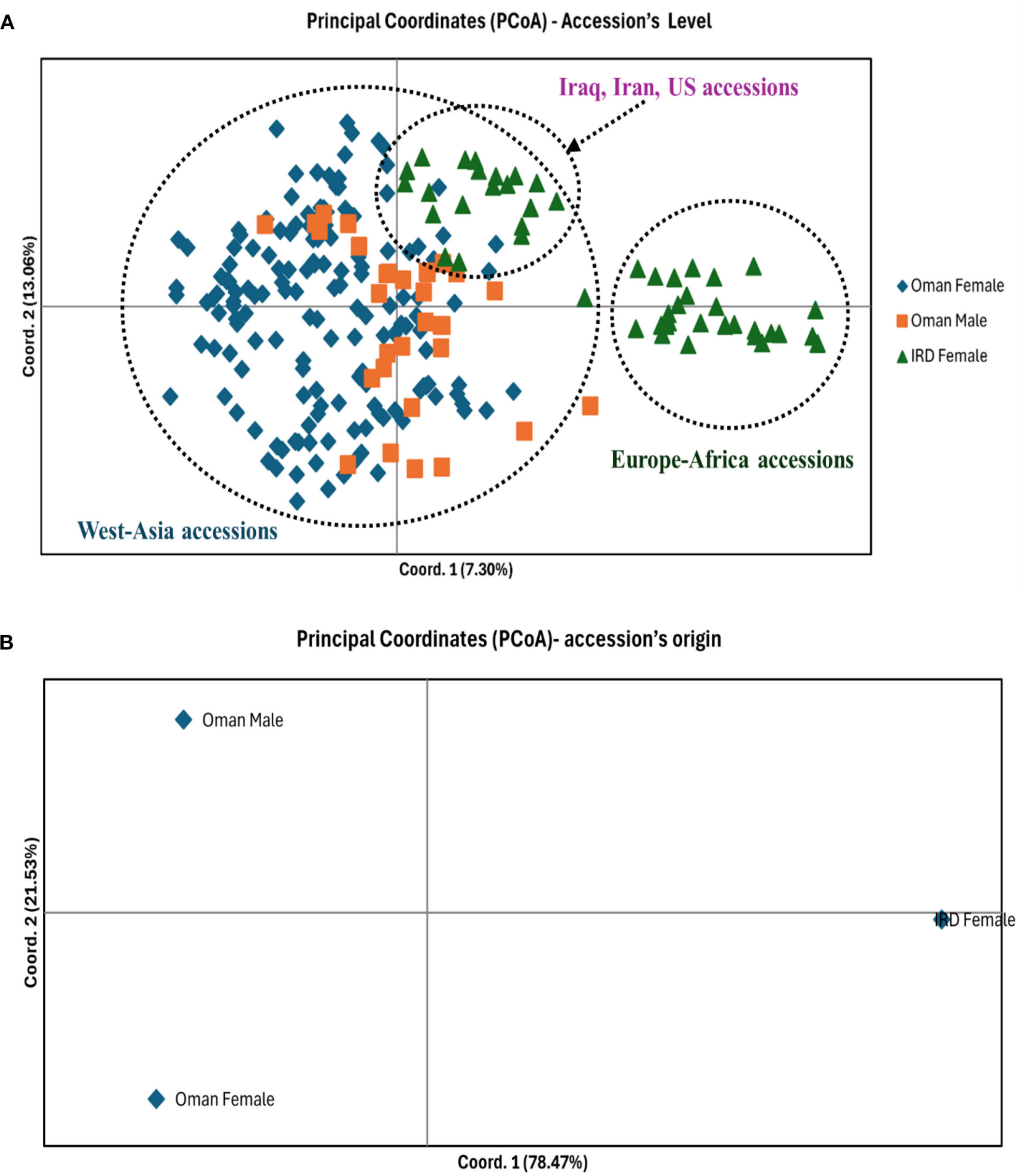
Principal Coordinates Analysis (PCoA) of date palm accessions based on 12 SSR loci. **(A)** Accession-level PCoA showing the distribution of Oman female (blue diamonds), Oman male (orange squares), and IRD female reference accessions (green triangles). **(B)** Origin-level PCoA based on group centroids.

Principal Coordinates Analysis (PCoA) based on 12 SSR loci revealed clear broad-scale patterns of genetic similarity among accessions, with separation driven primarily by the external IRD reference panel rather than by sex within the Omani collection (**Figure 1**). In the accession-level ordination, the first two axes explained a modest proportion of the total variation (Coord. 1 = 7.30%, Coord. 2 = 13.06%), reflecting the expected complexity of multi-locus microsatellite data while still resolving meaningful group structure.

Oman female and Oman male accessions showed substantial overlap in the PCoA space, clustering largely together within the main cloud of points. This indicates limited genetic differentiation between the Omani female and male groups at the marker set used, with no distinct sex-specific clustering evident. A small number of Omani male points extended toward the center-right of the plot, but these did not form a separate cluster and remained broadly embedded within the wider Omani distribution.

In contrast, IRD female accessions showed clearer separation from the Omani cluster, with two visible groupings. One IRD grouping formed a distinct cluster on the positive side of Coord. 1 (annotated as Europe–Africa accessions), indicating greater divergence from the Omani accessions. A second IRD subset clustered nearer the upper central region (annotated as Iraq, Iran, US accessions), positioned closer to the main Omani cluster, suggesting comparatively higher affinity to the West Asian gene pool. These trends were reinforced in the origin-level PCoA, where Coord. 1 (78.47%) separated IRD females from Omani groups, while Coord. 2 (21.53%) contributed to the separation between Oman female and Oman male centroids, though the overall pattern remained consistent with close relatedness within Oman.

### Neighbor-joining cluster analysis based on 12 SSR loci

**Figure 2:**
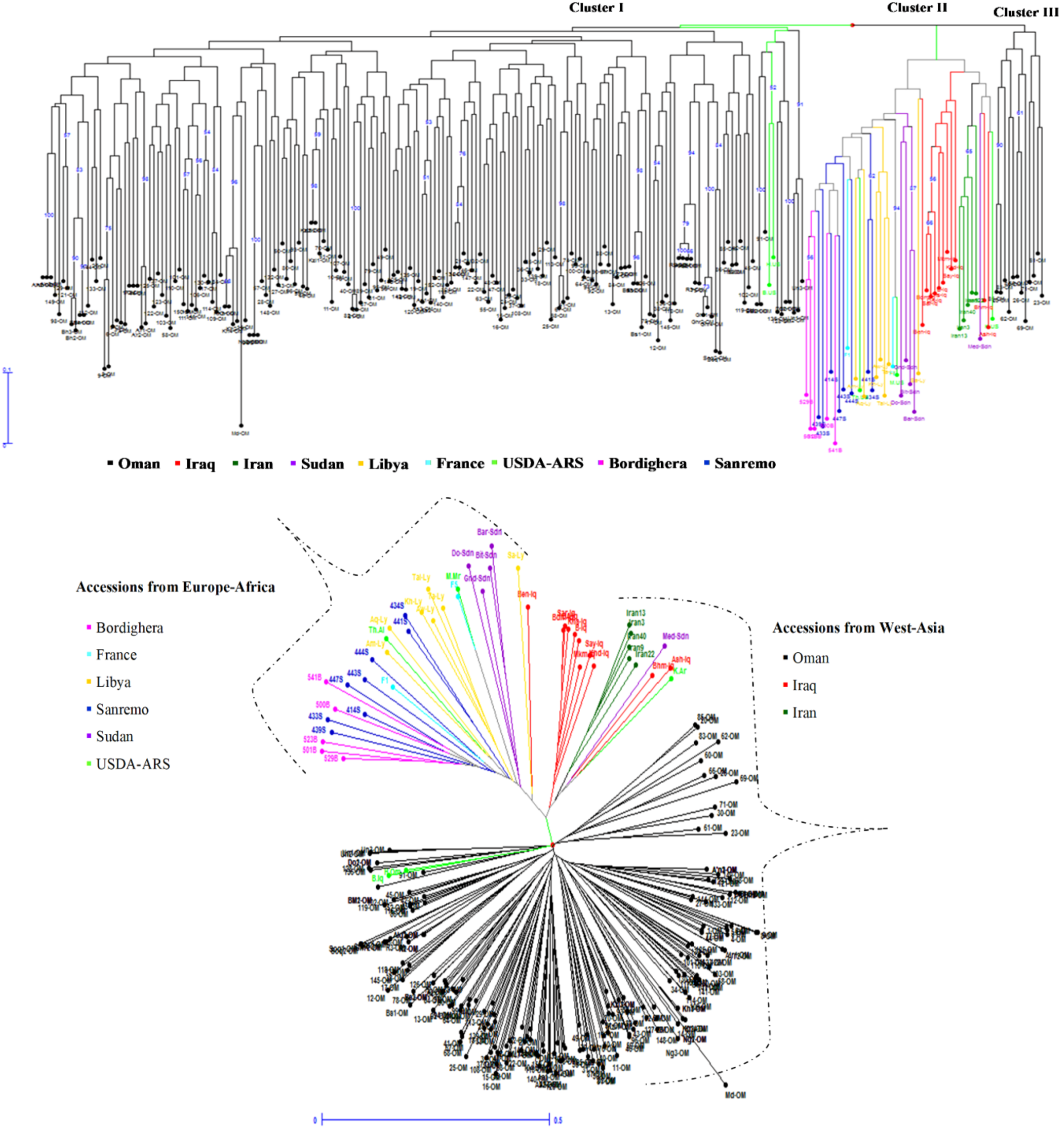
Neighbor-joining (NJ) clustering of date palm accessions based on genetic distances from 12 SSR loci. **(A)** NJ dendrogram showing relationships among accessions, with branches colored by country/collection of origin (Oman, Iraq, Iran, Sudan, Libya, France, USDA–ARS, Bordighera, and Sanremo). Three major groupings are indicated (Clusters I–III). **(B)** Radial representation of the same NJ tree highlighting broad geographic affinities, with accessions broadly separating into West-Asia–associated (Oman, Iraq, Iran) and Europe–Africa–associated (e.g., Bordighera, France, Libya, Sanremo, Sudan, USDA–ARS) groupings.

Neighbor-joining (NJ) clustering based on genetic distances from 12 SSR markers resolved the sampled date palm accessions into three main clusters (Clusters I–III), with a clear broad-scale separation that was largely consistent with geographic origin (**Figure 2**). The largest portion of the tree comprised predominantly Omani accessions, which grouped together across a broad, shallowly structured assemblage, indicating overall genetic similarity within the Omani collection while still retaining internal sub-branching among individual accessions.

A distinct pattern emerged for the external reference accessions, which formed more clearly differentiated groupings relative to Oman. Accessions annotated as originating from Europe–Africa sources (e.g., France, Libya, Sudan and related collections) tended to cluster together in a more compact section of the tree, suggesting closer genetic affinity among these non-Omani accessions compared with their relationship to the Omani set. In contrast, reference accessions associated with West Asia (e.g., Iraq and Iran) clustered nearer to the main Omani assemblage, indicating comparatively stronger genetic affinity with the Omani gene pool than the Europe–Africa group.

Overall, the NJ topology supports a primary division between an Oman/West-Asia–associated grouping and a more divergent Europe–Africa-associated grouping, while also indicating that most diversity is expressed as variation among accessions within the major Omani cluster rather than as deep splits among multiple strongly separated Omani subpopulations.

### Population structure analysis

**Figure 3:**
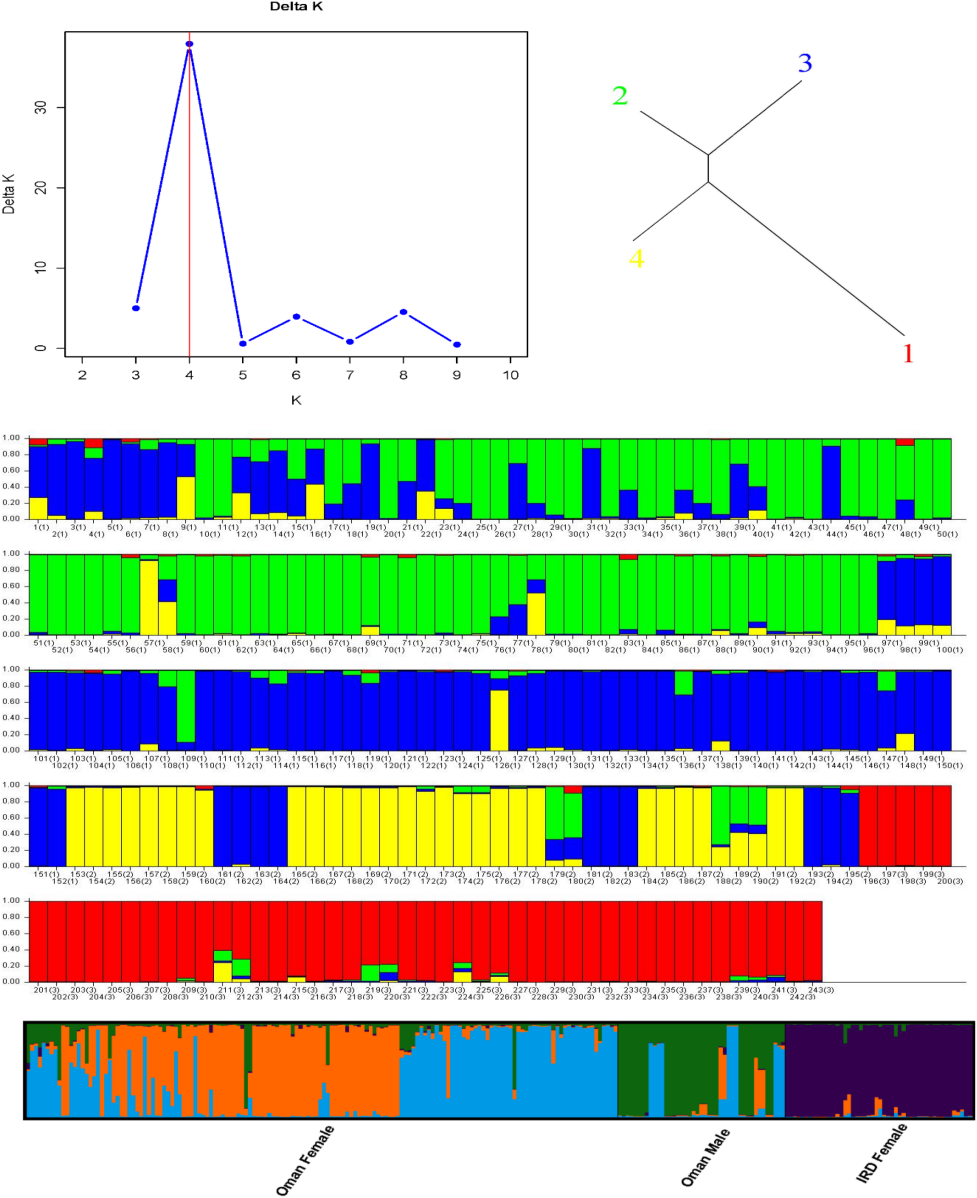
Population structure analysis of date palm accessions based on 12 SSR loci. Top left: ΔK plot (Evanno method) showing the highest support for K = 4. Top right: schematic clustering relationship among inferred groups. Middle panels: STRUCTURE bar plots for individual membership coefficients at K = 4, where each vertical bar represents one accession and colors represent inferred ancestry proportions; numbers in brackets indicate group identity with accessions ordered as Oman female (1), Oman male (2), and IRD (3). Bottom panel: summarized membership profiles grouped by origin/sex.

Population structure analysis identified a clear genetic subdivision among the sampled date palm accessions, with the strongest support for four ancestral clusters (K = 4) (**Figure 3**). The ΔK profile showed a pronounced peak at K = 4, indicating this value best captured the major structure present in the dataset relative to alternative K values tested.

At K = 4, individual assignment profiles revealed contrasting membership patterns among the study groups. Oman female accessions displayed the most heterogeneous composition, with individuals frequently showing mixed membership across multiple clusters, consistent with a broader range of genetic backgrounds within this group. In contrast, Oman male accessions were characterized by dominant membership in a single cluster for most individuals, with only limited admixture observed in a smaller subset, suggesting comparatively greater genetic homogeneity at the scale resolved by the 12 SSR loci. The IRD female reference panel showed the most distinct structure, with a large proportion of individuals assigned predominantly to a separate cluster that was uncommon in the Omani groups, indicating a clearer differentiation of the reference panel from the Oman gene pool under the inferred model.

Across the full dataset, admixture was not uniform. Some accessions exhibited near-complete membership in one cluster, whereas others showed substantial proportional contributions from two or more clusters. When individuals were viewed by group, the overall pattern supported close affinity between Oman females and Oman males (shared cluster components), while the IRD females were more frequently associated with a distinct cluster and showed a sharper transition in membership profiles. Together, these results indicate that the primary structure is driven by between-group differentiation involving the IRD reference panel, while sex-based separation within Oman is comparatively weak, and most Omani individuals retain evidence of shared ancestry and varying degrees of admixture.

## Discussion

### SSR marker polymorphism and genetic diversity summary

Microsatellite genotyping is well suited for analyzing genetically diverse, clonally propagated perennial crops such as date palm because SSRs are abundant, multi-allelic, and co-dominant (Holton, 2001). Across the 12 loci, the panel displayed substantial polymorphism, with a mean allelic richness of Na = 21, supporting broad diversity within the analyzed germplasm. Similar multi-allelic behavior has been reported in other date palm SSR studies, although allele counts vary markedly with sampling breadth and panel composition (Elshibli and Korpelainen, 2008; Chaluvadi et al., 2019; Elmeer and Mattat, 2015; Arabnezhad et al., 2012).

Heterozygosity estimates were generally high across loci, with locus-specific departures from equilibrium patterns, consistent with previous date palm reports (Metoui et al., 2017; Al-Faifi et al., 2016). Such variation between observed and expected heterozygosity can reflect biological and management factors, including seed-derived plants, mixed-pollen fertilization, and long-term movement of propagules and offshoots among growers (Bodian et al., 2014; Elmeer and Mattat, 2015), and broadly aligns with the range of SSR diversity patterns reported in other perennial crops and date palm datasets (Billotte et al., 2004).

Marker Informativeness was high overall (mean PIC = 0.781), with 11/12 loci exceeding the ≥0.5 threshold for informative SSRs, comparable to or higher than values reported previously (Zehdi et al., 2012; Chaluvadi et al., 2019). Fixation indices indicated low-to-moderate departures from random expectations and modest differentiation, consistent with population structure effects and sampling across multiple origins (Wright, 1965). The Shannon index was moderate and comparable to earlier work (Ezebilo et al., 2013), while significant HWE deviations at a subset of loci were consistent with prior SSR studies and can arise from mutation, sampling, or inbreeding-related processes (Akkak et al., 2009). The AMOVA pattern (higher within- than among-population variance) is also typical of long-lived, predominantly outcrossing tree crops (Perera et al., 2001). Finally, multivariate and clustering results supported broad-scale genetic grouping consistent with previously reported regional structure in *P. dactylifera* (Arabnezhad et al., 2012; Zehdi et al., 2012).

### Diversity patterns within Oman and sex-specific structure

Oman female and Oman male accessions exhibited broadly similar levels of genetic diversity, with only modest differences in mean allelic richness and heterozygosity. The pairwise population comparisons further support this where the lowest differentiation was between Oman female and Oman male (Fst = 0.021; Nei’s *D* = 0.143), indicating that sex-based subdivision within Oman is weak at the resolution provided by these 12 SSR loci. This pattern is consistent with expectations for a dioecious, obligately outcrossing crop in which gene flow is shaped by pollen movement and long-term propagation histories, leading to extensive sharing of genetic background across sex classes within the same cultivation system.

The STRUCTURE results were also concordant with this interpretation. Although ΔK supported K = 4, the Omani accessions did not segregate into sex-specific clusters; instead, Oman female individuals tended to show more heterogeneous membership profiles (greater admixture), while Oman male individuals more often displayed a dominant ancestry component with limited admixture in a subset. Importantly, these are differences in assignment patterns rather than strong sex-based separation, and they should be interpreted conservatively given that SSR-based ancestry estimates can be influenced by uneven sample sizes and allele frequency distributions across groups (Pritchard et al., 2000; Evanno et al., 2005).

### Differentiation between Oman and the IRD reference panel

The results support a clear but modest separation between Oman and IRD accessions. Pairwise estimates showed higher differentiation between Oman groups and IRD (Fst ≈ 0.057–0.059; Nei’s *D* ≈ 0.508–0.534) than between the two Omani sex groups, indicating that the principal axis of structure is associated with between-panel divergence (Oman *vs* IRD) rather than sex within Oman. This conclusion is consistent with the AMOVA, which partitioned 92% of variance within populations and 8% among populations, reflecting a genetic system where most diversity is maintained within groups but measurable structure exists at the population level (Excoffier et al., 1992). The scale of among group variance is also consistent with the modest Fst values observed, which fall within ranges typically interpreted as low-to-moderate differentiation in outcrossing species (Wright, 1965).

### Concordance across ordination and tree based clustering

Multiple complementary analyses converged on the same broad structure. The PCoA at the accession level showed substantial overlap between Oman female and Oman male accessions, reinforcing their close genetic affinity. IRD accessions formed more distinct groupings, including a clearly separated cluster and a second subset positioned closer to the Oman cloud, consistent with heterogeneous origins within IRD. The neighbor-joining clustering similarly recovered a topology where most Omani accessions formed a large assemblage with shallow internal structuring, while non-Omani reference accessions showed clearer grouping patterns, broadly consistent with West Asia *vs* Europe–Africa affinities. Such east–west structuring has been repeatedly reported in broader date palm datasets and is often interpreted as reflecting domestication history and long-term gene flow between regional gene pools (Zehdi-Azouzi et al., 2015; Rahman et al., 2022).

### Implications for germplasm management and breeding

The SSR panel showed high Informativeness (PIC) and substantial allelic diversity, supporting its use for accession fingerprinting, detection of duplicates/mislabeling, and routine germplasm curation. The weak differentiation between sex-defined groups indicates broadly shared genetic backgrounds, so conservation and breeding can treat male and female accessions as part of a common gene pool at this marker resolution. Practically, the diversity captured by these loci can help priorities genetically distinct parents (e.g., based on distance or cluster membership) to maximize allelic breadth in controlled crosses.

At the applied level, the weak sex-associated differentiation at this SSR resolution suggests that female cultivars and male trees can be managed as a broadly shared gene pool for routine curation, while still prioritizing the conservation of genetically distinct male genotypes that serve as pollen donors. Therefore, conservation sampling should focus on maximizing allelic diversity across clusters rather than sampling males and females as separate genetic units. In breeding and controlled pollination, SSR fingerprints can be used to select genetically distant male–female parent combinations to increase genetic breadth and reduce redundancy.

### Study limitations and future directions

Because the external accessions represent a limited, uneven reference panel, patterns involving that set should be interpreted as contextual affinities rather than country-level rankings of diversity. Additionally, uneven group sizes and heterogeneous origins within the IRD reference set may influence allele-frequency-based clustering. For this reason, STRUCTURE results are treated as descriptive and interpreted primarily through agreement with ordination (PCoA), distance-based clustering, and differentiation statistics. SSR-based structure can also be influenced by sampling design and locus-specific effects (including potential null alleles), which may contribute to heterozygote deficits at some loci. Future work should expand and balance sampling across regions (including more males) and apply higher-density SNP genotyping linked to standardized phenotypes to refine structure and strengthen breeding-relevant inference. Additionally, uneven group sizes and heterogeneous origins within the IRD reference set may influence allele-frequency-based clustering. For this reason, STRUCTURE results are treated as descriptive and interpreted primarily through agreement with ordination (PCoA), distance-based clustering, and differentiation statistics.

Overall, the 12 SSR markers revealed substantial genetic diversity within the Omani date palm collection, with Oman female and Oman male accessions showing closely shared genetic backgrounds and only weak sex-based differentiation. In contrast, the IRD reference panel was more clearly separated, providing useful external context without implying a balanced global comparison. Taken together, these results underline that most variation is maintained within populations, reinforcing the value of Oman’s germplasm for conservation, cultivar authentication, and informed parent selection in breeding programs.

## Conclusions

Using 12 SSR markers, this study provides a sex-stratified assessment of genetic diversity and structure in an Omani date palm collection, interpreted with a limited IRD reference panel to contextualize broader affinities without implying balanced global comparisons. The results show high within-group diversity across Oman female and Oman male accessions, alongside a consistent heterozygote deficit (Ho < He; positive Fis), and minimal differentiation between sexes within Oman (low Fst and Nei’s genetic distance). In contrast, comparisons with the IRD panel revealed modestly higher divergence, and structure analyses (K = 4), PCoA patterns, and the AMOVA partitioning (92% within; 8% among) collectively indicate that the principal genetic signal reflects between-panel differentiation rather than strong subdivision within Oman by sex.

These findings support the value of the Omani collection as a genetically diverse resource for conservation, cultivar authentication, and breeding, while emphasizing that inferences about geographic differences beyond Oman should remain conservative given the limited, unbalanced nature of the reference panel. Future work should expand external sampling, increase representation of male germplasm across regions, and incorporate higher-density SNP datasets to refine admixture estimates and support more detailed demographic and breeding-oriented analyses.

## Data Availability

The original contributions presented in the study are included in the article/**Supplementary Material**. Further inquiries can be directed to the corresponding authors.
