# Supplementary material for: Genetic diversity and population structure of Omani date palm assessed by SSR markers
Source: Front Plant Sci. 2026 Mar 2;17:1776708. doi: 10.3389/fpls.2026.1776708 (PMC12989608; doi:10.3389/fpls.2026.1776708)
Supplement: Supplementary file 1 [file Table1.docx]

**Supplementary Table 1.** List of 242 date palm accessions including male and female collected from Oman, Italy (Sanremo, Bordighera), USDA-ARS, France, Iraq, Libya, Sudan and Iran, their laboratory code and country of origin. (-) indicates accession name was not available.

| SN | Lab Code | Accession Name | Country of Origin | Sex |
| --- | --- | --- | --- | --- |
| 1 | OM01 | Qash Bunaringa | Oman | Female |
| 2 | OM02 | Miznag Ahmar | Oman | Female |
| 3 | OM03 | Qash Tabaq | Oman | Female |
| 4 | OM04 | Fard | Oman | Female |
| 5 | OM05 | Khsab | Oman | Female |
| 6 | OM06 | Qash Hagr | Oman | Female |
| 7 | OM07 | Qash Beladsait | Oman | Female |
| 8 | OM08 | Khinaizi Halaw | Oman | Female |
| 9 | OM09 | Hilali Hassa | Oman | Female |
| 10 | OM10 | Zabad | Oman | Female |
| 11 | OM11 | Qash Na’im | Oman | Female |
| 12 | OM12 | Qash Halaw | Oman | Female |
| 13 | OM13 | Qash Bu Rashid | Oman | Female |
| 14 | OM14 | Bentaami | Oman | Female |
| 15 | OM15 | Farid | Oman | Female |
| 16 | OM16 | Battash | Oman | Female |
| 17 | OM17 | Khinaizi Arabi | Oman | Female |
| 18 | OM18 | Qash Qaroot | Oman | Female |
| 19 | OM19 | Kalbi | Oman | Female |
| 20 | OM20 | Umm Alssila | Oman | Female |
| 21 | OM21 | Mazrm | Oman | Female |
| 22 | OM22 | Minaz | Oman | Female |
| 23 | OM23 | Mahlabbi | Oman | Female |
| 24 | OM24 | Khamri | Oman | Female |
| 25 | OM25 | Shabroot | Oman | Female |
| 26 | OM26 | Mekhildi | Oman | Female |
| 27 | OM27 | Nashu Ba’oodh | Oman | Female |
| 28 | OM28 | Mawaz | Oman | Female |
| 29 | OM29 | Nashu Ghoson | Oman | Female |
| 30 | OM30 | Qash Suwaih | Oman | Female |
| 31 | OM31 | Qash Qataari | Oman | Female |
| 32 | OM32 | Qash Ain Al Bakar | Oman | Female |
| 33 | OM33 | Qash Al Teeb | Oman | Female |
| 34 | OM34 | Qash Hareer | Oman | Female |
| 35 | OM35 | Hilali Ahmer | Oman | Female |
| 36 | OM36 | Qash Zamel | Oman | Female |
| 37 | OM37 | Ghrabo | Oman | Female |
| 38 | OM38 | Shaeeri | Oman | Female |
| 39 | OM39 | Qash Abu Keebal | Oman | Female |
| 40 | OM40 | Bayadh | Oman | Female |
| 41 | OM41 | Qash Mushrab | Oman | Female |
| 42 | OM42 | Qash Ghssan | Oman | Female |
| 43 | OM43 | Qash Hmdan | Oman | Female |
| 44 | OM44 | Qash Habeeb | Oman | Female |
| 45 | OM45 | Qash Abu Saif | Oman | Female |
| 46 | OM46 | Qash Habisha | Oman | Female |
| 47 | OM47 | Hessas | Oman | Female |
| 48 | OM48 | Qash Manzef | Oman | Female |
| 49 | OM49 | Zaad | Oman | Female |
| 50 | OM50 | Naghal | Oman | Female |
| 51 | OM51 | Qash Qantara | Oman | Female |
| 52 | OM52 | Ma’an | Oman | Female |
| 53 | OM53 | Bunaringa | Oman | Female |
| 54 | OM54 | Jebri | Oman | Female |
| 55 | OM55 | Hilali Makran | Oman | Female |
| 56 | OM56 | Qash Humaid bin Ghareb | Oman | Female |
| 57 | OM57 | Menhi | Oman | Female |
| 58 | OM58 | Mebseli | Oman | Female |
| 59 | OM59 | Bershi | Oman | Female |
| 60 | OM60 | Rees | Oman | Female |
| 61 | OM61 | Qash Al Hareem | Oman | Female |
| 62 | OM62 | Nashu Ewan | Oman | Female |
| 63 | OM63 | Malkt Deeni | Oman | Female |
| 64 | OM64 | Mayasi | Oman | Female |
| 65 | OM65 | Hadaqi | Oman | Female |
| 66 | OM66 | Abu Qareen | Oman | Female |
| 67 | OM67 | Selahni | Oman | Female |
| 68 | OM68 | Qash Sahrh | Oman | Female |
| 69 | OM69 | Naboot Saif | Oman | Female |
| 70 | OM70 | Qash Al Yamam | Oman | Female |
| 71 | OM71 | Qash Humaid | Oman | Female |
| 72 | OM72 | Naghl Lulu | Oman | Female |
| 73 | OM73 | Qash Al Rabeea | Oman | Female |
| 74 | OM74 | Mazni | Oman | Female |
| 75 | OM75 | Qash Al wa'b | Oman | Female |
| 76 | OM76 | Qash Al Saghiay | Oman | Female |
| 77 | OM77 | Abu Al Audooq | Oman | Female |
| 78 | OM78 | Qash Shafer | Oman | Female |
| 79 | OM79 | Alak | Oman | Female |
| 80 | OM80 | Snah | Oman | Female |
| 81 | OM81 | Qash Hatami | Oman | Female |
| 82 | OM82 | Qash Katerh | Oman | Female |
| 83 | OM83 | Qash Al Rabab | Oman | Female |
| 84 | OM84 | Qash Sba | Oman | Female |
| 85 | OM85 | Qash Hiyshmi | Oman | Female |
| 86 | OM86 | Qash Al Teebi | Oman | Female |
| 87 | OM87 | Qash Rsheed | Oman | Female |
| 88 | OM88 | Nashu Al khzma | Oman | Female |
| 89 | OM89 | Berzeban | Oman | Female |
| 90 | OM90 | Nashu Al Wakhrh | Oman | Female |
| 91 | OM91 | Naghlt Khalas | Oman | Female |
| 92 | OM92 | Khalas Oman | Oman | Female |
| 93 | OM93 | Qash Gha'roof | Oman | Female |
| 94 | OM94 | Qash Ghafan | Oman | Female |
| 95 | OM95 | Qash Nas'rah | Oman | Female |
| 96 | OM96 | Qash Gheniyah | Oman | Female |
| 97 | OM97 | Qash Nwaihi | Oman | Female |
| 98 | OM98 | Qash Al Masbt | Oman | Female |
| 99 | OM99 | Qasht Naghal | Oman | Female |
| 100 | OM100 | Qash Ali | Oman | Female |
| 101 | OM101 | Qash Hareb | Oman | Female |
| 102 | OM102 | Qash Nasir | Oman | Female |
| 103 | OM103 | Qash Safiyh | Oman | Female |
| 104 | OM104 | Qash Fakhrh | Oman | Female |
| 105 | OM105 | Qash Suwaid | Oman | Female |
| 106 | OM106 | Qash Ba'Omar | Oman | Female |
| 107 | OM107 | Nashu Shamiss | Oman | Female |
| 108 | OM108 | Shahl | Oman | Female |
| 109 | OM109 | Ramli | Oman | Female |
| 110 | OM110 | Shiham | Oman | Female |
| 111 | OM111 | Seedi | Oman | Female |
| 112 | OM112 | Khashkar | Oman | Female |
| 113 | OM113 | Nashu Saleh | Oman | Female |
| 114 | OM114 | Nashu Maneh | Oman | Female |
| 115 | OM115 | Lulu | Oman | Female |
| 116 | OM116 | Rabai | Oman | Female |
| 117 | OM117 | Qash Suwailim | Oman | Female |
| 118 | OM118 | Bata | Oman | Female |
| 119 | OM119 | Barny | Oman | Female |
| 120 | OM120 | Nashu Fahood | Oman | Female |
| 121 | OM121 | Muttrahi | Oman | Female |
| 122 | OM122 | Bidaa | Oman | Female |
| 123 | OM123 | Medairki | Oman | Female |
| 124 | OM124 | Kibkab | Oman | Female |
| 125 | OM125 | Huzaifah | Oman | Female |
| 126 | OM126 | Nashu Al Khashiyah | Oman | Female |
| 127 | OM127 | Hawam | Oman | Female |
| 128 | OM128 | Qadmi | Oman | Female |
| 129 | OM129 | Qash Gammah | Oman | Female |
| 130 | OM130 | Qash Saima | Oman | Female |
| 131 | OM131 | Medlooki | Oman | Female |
| 132 | OM132 | Damoos | Oman | Female |
| 133 | OM133 | Qash Hareez | Oman | Female |
| 134 | OM134 | Qash Al Looz | Oman | Female |
| 135 | OM135 | Qash Al Semnah | Oman | Female |
| 136 | OM136 | Qash Hamreiyah | Oman | Female |
| 137 | OM137 | Qash Baloobiya | Oman | Female |
| 138 | OM138 | Qash Abu Al Sohoon | Oman | Female |
| 139 | OM139 | Qash Mishah | Oman | Female |
| 140 | OM140 | Qash Al Dahiyah | Oman | Female |
| 141 | OM141 | Qash Al Ramliyah | Oman | Female |
| 142 | OM142 | Qash Al Wali | Oman | Female |
| 143 | OM143 | Medgahdel | Oman | Female |
| 144 | OM144 | Bel'aq | Oman | Female |
| 145 | OM145 | Jebreen | Oman | Female |
| 146 | OM146 | Qash Bussemen | Oman | Female |
| 147 | OM147 | Hilali Omani | Oman | Female |
| 148 | OM148 | Qash Ghinuwi | Oman | Female |
| 149 | OM149 | Qash A'Saba Al Aruz | Oman | Female |
| 150 | OM150 | Serna | Oman | Female |
| 151 | OM151 | Khalas Al Zahra | Oman | Female |
| 152 | OM152 | Khori 1 | Oman | Male |
| 153 | OM153 | Khori 2 | Oman | Male |
| 154 | OM154 | Khori 3 | Oman | Male |
| 155 | OM155 | Khori 4 | Oman | Male |
| 156 | OM156 | Naghayli 1 | Oman | Male |
| 157 | OM157 | Naghayli 2 | Oman | Male |
| 158 | OM158 | Naghayli 3 | Oman | Male |
| 159 | OM159 | Medgahdel | Oman | Male |
| 160 | OM160 | Bahlani 1 | Oman | Male |
| 161 | OM161 | Bahlani 2 | Oman | Male |
| 162 | OM162 | Bahlani 3 | Oman | Male |
| 163 | OM163 | Bahlani 4 | Oman | Male |
| 164 | OM164 | Ghareef 1 | Oman | Male |
| 165 | OM165 | Ghareef 2 | Oman | Male |
| 166 | OM166 | Ghareef 4 | Oman | Male |
| 167 | OM167 | Al Fahel Al dhakm 1 | Oman | Male |
| 168 | OM168 | Al Fahel Al dhakm 2 | Oman | Male |
| 169 | OM169 | Unknown Male 1 | Oman | Male |
| 170 | OM170 | Unknown Male 2 | Oman | Male |
| 171 | OM171 | Unknown Male 3 | Oman | Male |
| 172 | OM172 | Bu'Sab'ah 1 | Oman | Male |
| 173 | OM173 | Bu'Sab'ah 2 | Oman | Male |
| 174 | OM174 | Bu'Sab'ah 3 | Oman | Male |
| 175 | OM175 | Rghad 1 | Oman | Male |
| 176 | OM176 | Rghad 2 | Oman | Male |
| 177 | OM177 | Rghad 3 | Oman | Male |
| 178 | OM178 | A'reesh 1 | Oman | Male |
| 179 | OM179 | A'reesh 2 | Oman | Male |
| 180 | OM180 | An'bati 1 | Oman | Male |
| 181 | OM181 | An'bati 2 | Oman | Male |
| 182 | OM182 | An'bati 3 | Oman | Male |
| 183 | OM183 | Al Maquidha 1 | Oman | Male |
| 184 | OM184 | Al Maquidha 2 | Oman | Male |
| 185 | OM185 | Soo'qum 1 | Oman | Male |
| 186 | OM186 | Soo'qum 2 | Oman | Male |
| 187 | OM187 | Khzini 1 | Oman | Male |
| 188 | OM188 | Khzini 2 | Oman | Male |
| 189 | OM189 | Khzini 3 | Oman | Male |
| 190 | OM190 | Do'wairah 1 | Oman | Male |
| 191 | OM191 | Do'wairah 2 | Oman | Male |
| 192 | OM192 | Al Lasah 1 | Oman | Male |
| 193 | OM193 | Al Lasah 2 | Oman | Male |
| 194 | OM194 | Al Lasah 3 | Oman | Male |
| 195 | 414 | - | Italy/Sanremo | Female |
| 196 | 433 | - | Italy/Sanremo | Female |
| 197 | 434 | - | Italy/Sanremo | Female |
| 198 | 439 | - | Italy/Sanremo | Female |
| 199 | 441 | - | Italy/Sanremo | Female |
| 200 | 443 | - | Italy/Sanremo | Female |
| 201 | 444 | - | Italy/Sanremo | Female |
| 202 | 447 | - | Italy/Sanremo | Female |
| 203 | 500 | - | Italy/Bordighera | Female |
| 204 | 501 | - | Italy/Bordighera | Female |
| 205 | 523 | - | Italy/Bordighera | Female |
| 206 | 529 | - | Italy/Bordighera | Female |
| 207 | 541 | - | Italy/Bordighera | Female |
| 208 | Khalas | Khalas | Arabia; USDA | Female |
| 209 | Thory | Thory | Algeria; USDA | Female |
| 210 | Hilali | Hilali | Oman; USDA | Female |
| 211 | Barhee | Barhee | Iraq; USDA | Female |
| 212 | Medjool | Medjool | Morocco; USDA | Female |
| 213 | Fran1 | - | France | Female |
| 214 | Fran5 | - | France | Female |
| 215 | DA-Iq | Daml Asfer | Iraq | Female |
| 216 | B-Iq | Badmi | Iraq | Female |
| 217 | Sar-Iq | Sarmadti | Iraq | Female |
| 218 | Mkm-Iq | Maktoom | Iraq | Female |
| 219 | Bdm-Iq | Bdmalki | Iraq | Female |
| 220 | Ben-Iq | Benosh | Iraq | Female |
| 221 | Ash-Iq | Ashrasi | Iraq | Female |
| 222 | Khs-Iq | Khastawi | Iraq | Female |
| 223 | Say-Iq | Saylani | Iraq | Female |
| 224 | Bhm-Iq | Bahram | Iraq | Female |
| 225 | Khd-Iq | Khadrawy | Iraq | Female |
| 226 | Aw-Ly | Awreeq | Libya | Female |
| 227 | Kh-Ly | Khmag | Libya | Female |
| 228 | Ta-Ly | Taghiyat | Libya | Female |
| 229 | Am-Ly | Amreer | Libya | Female |
| 230 | Tal-Ly | Talees | Libya | Female |
| 231 | Sa-Ly | Saidi | Libya | Female |
| 232 | Aq-Ly | Aqudool | Libya | Female |
| 233 | Med-Sdn | Medina | Sudan | Female |
| 234 | Gnd-Sdn | Gondaila | Sudan | Female |
| 235 | Bar-Sdn | Barakawi | Sudan | Female |
| 236 | Bit-Sdn | Bitamoda | Sudan | Female |
| 237 | Do-Sdn | Dogna | Sudan | Female |
| 238 | Iran3 | Bentossbae | Iran | Female |
| 239 | Iran9 | Gentaar | Iran | Female |
| 240 | Iran13 | Zahedi | Iran | Female |
| 241 | Iran22 | Soweidance | Iran | Female |
| 242 | Iran40 | Halilehei | Iran | Female |
